# Experimental investigation and numerical modelling of photocurrent oscillations in lattice matched Ga_1−*x*_In_*x*_N_*y*_As_1−*y*_/GaAs quantum well p-i-n photodiodes

**DOI:** 10.1186/1556-276X-9-84

**Published:** 2014-02-18

**Authors:** Ben Royall, Hagir Khalil, Simone Mazzucato, Ayse Erol, Naci Balkan

**Affiliations:** 1 Department of Physics and Astronomy, Hicks Building, Hounsfield Road, Sheffield S3 7RH, UK; 2 School of CSEE, University of Essex, Colchester CO4 3SQ, UK; 3 LPCNO, INSA-UPS-CNRS, 135 av. de Rangueil, Toulouse 31077 CEDEX 4, France; 4 Department of Physics, Science Faculty, Istanbul University, Vezneciler, Istanbul 34134, Turkey

## Abstract

Photocurrent oscillations, observed at low temperatures in lattice-matched Ga_1−*x*_In_*x*_N_*y*_As_1−*y*_/GaAs multiple quantum well (MQW) p-i-n samples, are investigated as a function of applied bias and excitation wavelength and are modelled with the aid of semiconductor simulation software. The oscillations appear only at low temperatures and have the highest amplitude when the optical excitation energy is in resonance with the GaInNAs bandgap. They are explained in terms of electron accumulation and the formation of high-field domains in the GaInNAs QWs as a result of the disparity between the photoexcited electron and hole escape rates from the QWs. The application of the external bias results in the motion of the high-field domain towards the anode where the excess charge dissipates from the well adjacent to anode via tunnelling.

## Background

Since the first demonstration of the growth of dilute nitrides in the mid-1990s [1], research in the field has grown continuously as the vast number of publications, review papers and books indicate [2-4]. Among dilute nitrides, Ga_1−*x*_In_*x*_N_*y*_As_1−*y*_ is a quaternary material which can be grown lattice-matched to GaAs and be incorporated into GaAs-based distributed Bragg reflector structures (DBRs). Furthermore, since incorporation of just a few percent of nitrogen in GaInAs causes a large bandgap reduction in GaInNAs, this alloy can be employed for near-infrared applications. Over the last two decades, a number of optoelectronic devices based on this alloy, including emitters [5-7], detectors [8,9], solar cells [10,11], optical amplifiers [12,13] and saturable absorber mirrors [14] has been demonstrated successfully.

Compared to the major industrial competitors, the InP-based devices, GaInNAs/GaAs has a higher conduction band (CB) offset, which provides good electron confinement [15,16]. For applications as lasers in the telecom wavelengths of 1.3 μm, typical composition of Ga_1−*x*_In_*x*_N_*y*_As_1−*y*_ with *x* approximately 30% and *y* approximately 2% ensures also hole confinement, resulting in better temperature stability of the laser threshold current [17]. However, in applications as photodetectors and solar cells where the thickness of the dilute nitride layer has to be large for enhanced photon absorption, perfect lattice matching to GaAs is required and the relative In and N compositions have to be changed, usually in the ratio In:N equal to 3:1. This results in poor hole confinement compared to that of the electrons [3].

Dilute nitride-based semiconductors are widely used in solar cell applications because both the bandgap and lattice constant can be altered readily by adjusting the N and In contents. Consequently, when dilute nitride solar cells are used in lattice-matched multi-junction tandem cells, an improved coverage of solar spectrum and higher power efficiencies are achieved [18-20]. In a recent patented work, an efficient carrier collection [21] has been proposed, where the CB confinement energy and the barrier thickness are designed to favour sequential thermionic emission and resonant tunnelling of electrons. The ‘superlattice’ approach was also employed in transport [22] and QW infrared detector devices [23-25].

In this work, we use GaInNAs/GaAs multiple quantum wells (MQWs) in the intrinsic region of a GaAs p-i-n structure. The device photoresponse and photocurrent characteristics measured at low temperatures show clearly oscillations in the current–voltage (*I*-*V*) curves. The number of the oscillations corresponds to the number of the QWs in the intrinsic region as reported by us elsewhere [26,27]. In this paper, we aim to understand the underlying mechanisms for the observed oscillations via comparing our results with an extensive simulation model. The semiconductor simulation software, Simwindows32 [28], is used successfully to account for the experimental results.

## Methods

Four GaInNAs/GaAs MQW p-i-n photodiodes have been investigated in this work. They were grown by molecular beam epitaxy (MBE) on doped (100)-oriented GaAs substrates. The structural parameters of all the investigated samples are listed in Table 1. The In content of the QWs was kept to three times the N content to achieve lattice matching with the GaAs layers [29], and this was confirmed by XRD measurements. In sample AsN2604, the intrinsic region consists of 10 undoped GaInNAs QWs with thickness varying from 3.8 to 11 nm. The samples VN1585 and AsN3134 have 10 QWs with a constant well width of 10 nm. AsN3138 is almost identical to AsN3134 but with 20 QWs. In all samples, the wells are separated from each other by wide GaAs barriers. The samples were fabricated in the shape of a mesa structure, with a top circular aperture of 1 mm diameter. Further details about structure, growth parameters and fabrication process can be found elsewhere [19].

**Table 1:** Samples' key structure parameters together with the RT PL peak wavelength

**Sample**	**No. QWs**	**QW thickness (nm)**	***x *** **and *y *** **(%)**	**Structure**	**RT PL peak *λ *** **(nm)**
AsN2604	10	3.8 to 11	4 and 1.5	p-i-n	1,033
AsN3134	10	10	4.8 and 1.6	p-i-n	1,067
AsN3138	20	10	4.8 and 1.6	p-i-n	1,077
VN1585	10	10	3 and 1	n-i-p	998

Optical quality of the devices was determined using CW photoluminescence (PL) as a function of temperature. Table 1 lists the room temperature (RT) GaInNAs PL peak wavelengths.

The p-n junction quality was determined by measuring the current–voltage characteristic in the growth direction, in darkness, in the forward and reverse bias configurations. The measurements were carried out over the temperature range between *T* = 15 K and 300 K. Photocurrent oscillations were also carried out at the same temperature range when the samples were illuminated using a 950-nm LED. Spectral photoresponse was measured by uniformly illuminating the samples with variable wavelength monochromatic light.

## Results and discussion

**Figure 1:**
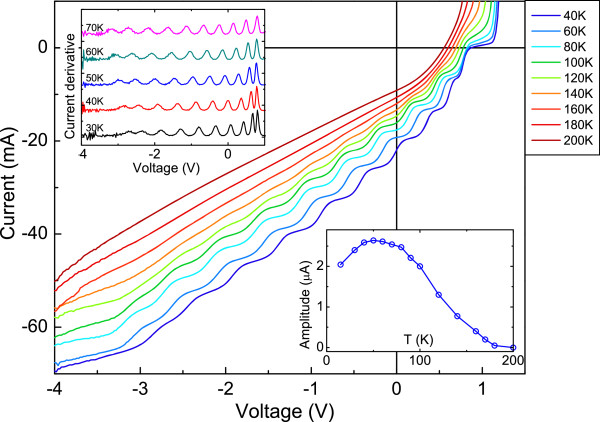
**VN1585 temperature-dependent *I*-*V *under illumination.** The top left inset shows the derivative of the *I*-*V* curves, while the right bottom one shows the oscillations' amplitude as a function of temperature.

Figure 1 shows the photocurrent versus voltage characteristics for sample VN1585 at temperatures between *T* = 40 K and 200 K. At *T* > 140 K, the curves are smooth at all the applied bias voltages. At *T* = 140 K, a number of small discrete steps appear, and at around *T* approximately 120 K, these steps are clearly visible and get increasingly more pronounced with decreasing temperature. The first derivatives of the *I*-*V* curves are plotted in the top left inset in Figure 1. It is clear that the steps in the photocurrent correspond to well-defined oscillations in the *dI*/*dV* curves. The number of the oscillations, 10, is the same as the number of QWs in the sample. The amplitude of each oscillation has the temperature dependence as shown in the bottom inset in Figure 1. All the samples studied showed similar behaviour to that in VN1585.

**Figure 2:**
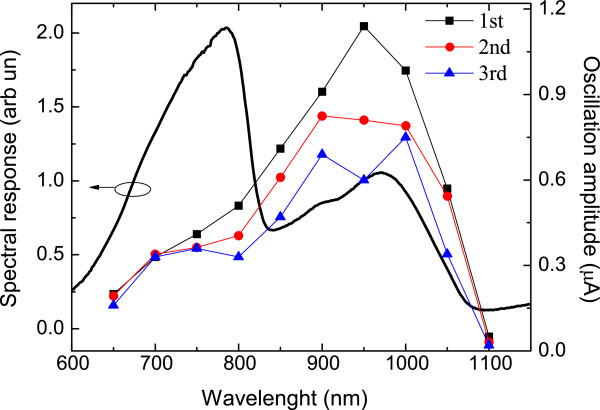
Comparison between spectral photoresponse of AsN2604 and amplitude of the first three oscillations versus excitation wavelength.

In order to establish whether the oscillations are associated with optically excited carriers in the GaInNAs QWs, the spectral dependence of the photocurrent were measured. The spectral response of AsN2604 (Figure 2) increases with increasing wavelength but cuts off at a wavelength of 830 nm corresponding to the GaAs bandgap. At wavelengths between 830 and 1,060 nm, the majority of incident photons are absorbed only in the GaInNAs QWs and there is a broad peak followed by a cutoff around 1,060 nm corresponding to the bandgap of GaInNAs quantum wells. In Figure 2, we also plotted the amplitudes of three different photocurrent (PC) oscillations versus the excitation wavelength. It is clear that the maximum amplitude of the oscillations is reached when the excitation wavelength is in resonance with the GaInNAs bandgap, confirming that they are associated with photogenerated carriers within the GaInNAs QWs.

**Figure 3:**
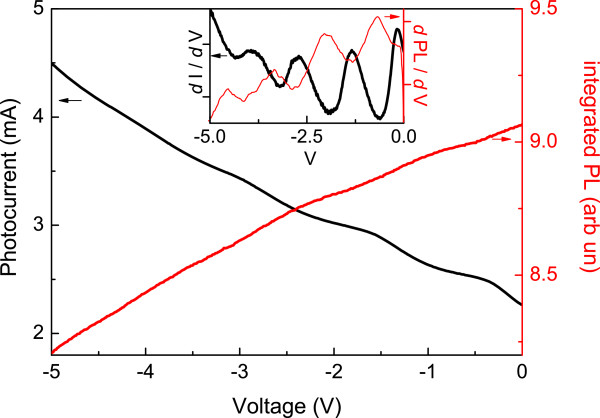
***I*-*V *and integrated PL versus applied voltage for AsN2604 at *T*** **= 100 K.** The derivatives of the curves are plotted in the inset.

Further evidence for the instabilities in PC being associated with photogenerated carriers in the QWs comes from the observation of PL oscillations when the device bias is varied [27]. In this experiment, the PL signal was integrated over all the GaInNAs optical transition. It is clear from Figure 3 that the PL oscillations are out of phase with the PC oscillations and occur at the same applied bias voltages. This is because when the oscillating component of the non-radiative current goes through a minimum, the radiative current will increase leading to the observed maximum in PL.

**Figure 4:**
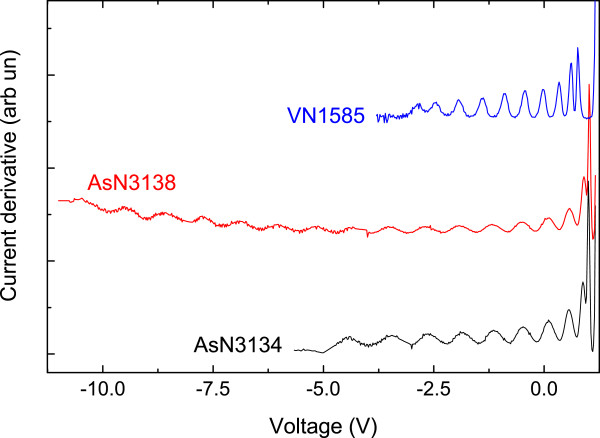
**First derivative of AsN3134, AsN3138 and VN1585 *I*-*V *curves at *T*** **= 15 K, shifted for clarity.**

The first derivatives of the *I*-*V* curves for VN1585, AsN3134 and AsN3138 are shown in Figure 4. The samples with 10 QWs, VN1585 and AsN3134 have 10 clear oscillations. In AsN3138 with 20 QWs, there are 18 distinct peaks in the PC. We were not able to observe the two further expected peaks in this sample because the diode entered the breakdown region.

The origin of these oscillations is to be searched into the different confinement of electrons and holes inside the GaInNAs QWs. Table 2 lists the CB offset Δ*E*_C_ and the valence band (VB) offset Δ*E*_V_, calculated using the band anti-crossing model and a 8-band **k.p** Hamiltonian [30]. Δ*E*_V_ is considerably smaller than Δ*E*_C_ for all samples, leading to good electron confinement but poor hole confinement. Because of the QW bidimensional structure, carriers will lay in a discrete number of subband energy levels, whose number will depend upon the thickness of the QW. In our samples at *T* = 100 K, up to three levels are allowed. Their energies (measured from the band edges) are also listed in Table 2. It can be noticed that some of them are so close to the band edges (few meV) that it will be very easy for the carriers there to escape into the surrounding barriers.

**Table 2:** Electron and hole confinement energies and band offsets

**Sample**	**Δ** ***E***_**C **_**(meV)**	**Electron confinement energies (meV)**	**Δ** ***E***_**V **_**(meV)**	**Hole confinement energies (meV)**
AsN2604 (for the 3.8-nm QW)	252	81, 250	20	11, 16
AsN2604 (for the 11-nm QW)		20, 78, 164		4, 9, 13
AsN3134 and AsN3138	270	24, 91, 187	23	4, 12, 15
VN1585	188	23, 87, 172	14	4, 9, 12

For AsN2604, in which the 10 QWs have different thickness, the energy levels are listed for the 3.8 and 11 nm only.

As a result, the photoexcited holes are readily thermionically excited out of the wells and swept out of the intrinsic region under the influence of the external and built-in electric field as we have reported elsewhere [31]. This is a very fast process and would give a fast component to the PC transients. The main contribution to the steady state PC is therefore due to the electrons. In order for an electron photogenerated in the QW to contribute to the photocurrent, it must either be thermionically excited or tunnel into the continuum over the CB discontinuity or sequentially tunnel into the neighbouring wells [23,32]. Which of these two processes dominates PC should depend upon the temperature, barrier height/thickness and the applied bias.

Under optical illumination, electron–hole pairs are generated in the quantum wells. The disparity between the electron and hole escape rates from the QWs means that even a small electric field across a well will allow the holes to escape. Instead, because of the different confinement energy, the electrons are trapped in the well, and without holes in the valence band, they cannot recombine and start accumulating. This electron accumulation acts as a space charge, screening the built-in charge of the junction. Consequently, the applied voltage is not uniformly distributed across the intrinsic region; instead, it will be applied only between the positive charge at the edge of the n-type region and the closest well with a large negative charge. High-field domain [22] is formed, and an increase in the applied bias leads to the reduction of the electron escape time for a single well at a time. Further increase of the electric field makes the high-field domain high enough to allow electrons to escape and flow the n-type region resulting in a sudden change (an oscillation) in PC.

PC oscillations are visible also in superlattice structures [24], but they are based to the strong carrier coupling among the wells, leading to the occurrence of negative differential resistance (NDR) via sequential resonant tunnelling between adjacent QWs. However, because of the thick GaAs barriers between adjacent QWs in our structures, sequential resonant tunnelling is unlikely to occur. Hence, we did not observe any NDR. Thermionic emission from the QWs and Fowler-Nordheim [33] tunnelling from the well adjacent to the n-type bulk region are instead the two likely electron escape mechanisms. The hole capture time by the QWs is much longer than the hole flight time between adjacent wells so that the holes transfer rapidly to the p-region of the device without being captured [31]. This results in the net negative charge accumulation in the wells. PC oscillations do not occur in samples with a strong hole confinement, i.e. in samples with high In concentration as implied by Chen et al. [34] where the indium concentration was 35% and the nitrogen 0.23%, with Δ*E*_C_ = 510 meV and Δ*E*_V_ = 130 meV.

### Modelling results

In order to support our explanation of the PC oscillations, we modelled the current–voltage curves of our devices using the semiconductor simulation package SimWindows32 [28]. This software is able to model carrier escape from the QWs mainly via thermionic emission by considering the lowest energy subband; nonetheless, it has been able to recreate the oscillations and helped improve our understanding of the mechanisms involved in our samples. SimWindows32 is fundamentally a 1D drift-diffusion simulator that solves Poisson's equation, the current continuity equations, the photon rate equation and the energy balance equation in steady state.

The simulation presented here refers to the device AsN3134, using the values present for GaAs in the Simwindows32 material parameter file and in the literature for GaInNAs [35-37]. The sample bandgap was taken from the PL measurements. Optical excitation was included in the simulation via monochromatic light at *λ* = 950 nm to excite only the GaInNAs/GaAs QWs, with a 10-mW/cm^2^ incident intensity. The band profile and the electron and hole carrier concentrations are recorded as a function of sample growth direction for a selection of applied voltages from 1.4 V down to −5 V. Temperature dependence of PC was simulated and showed that the oscillations are indeed absent at RT and start appearing when lowering the temperature below 200 K, in agreement with the experimental results. The following results refer to the case of *T* = 100 K, where the amplitude of the oscillations reaches its maximum (see bottom inset of Figure 1).

**Figure 5:**
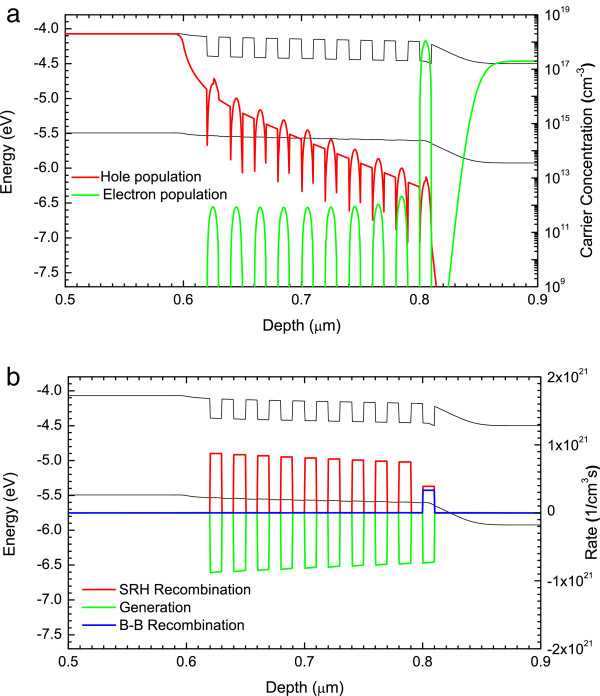
**Photocurrent- and photoconductance-voltage characteristics of AsN3134 at 100 K under 10 mW/cm**^**2 **^**illumination, modelled by Simwindows32.** The blue arrows indicate the points discussed in Figures 6 to 8.

The simulated *I*-*V* results under illumination and their derivative (conductance) are shown in Figure 5 and show the same features which were observed experimentally.

We can clearly see the 10 peaks corresponding to the 10 QWs, in the same way as shown in Figure 4. Throughout the following discussion, we will refer to the peaks from P1 to P10 with decreasing applied voltage, whereas the QWs will be called QW1 to QW10 going from the n- to the p-type region. The simulation results will show that carriers escaping from a specific QW will result in the corresponding number peak.

**Figure 6:**
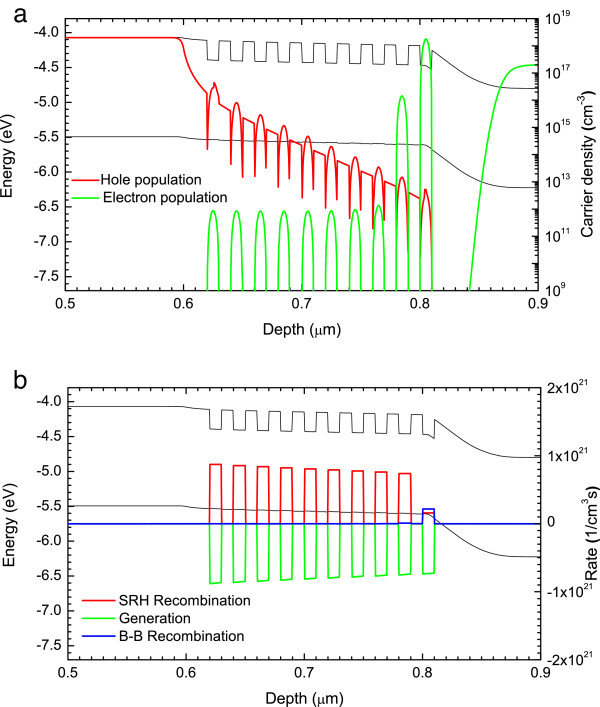
***V***_**app**_ **= 1 V.** Band profile and **(a)** electron/hole populations, **(b)** SRH, B-B recombination and optical generation rates.

**Figure 7:**
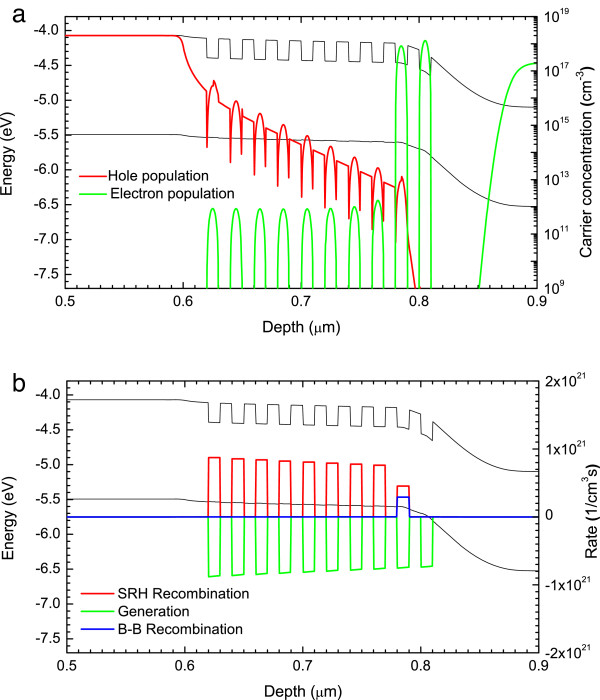
***V***_**app**_ **= 0.7 V.** Band profile and **(a)** electron/hole populations, **(b)** SRH, B-B recombination and optical generation rates.

**Figure 8:**
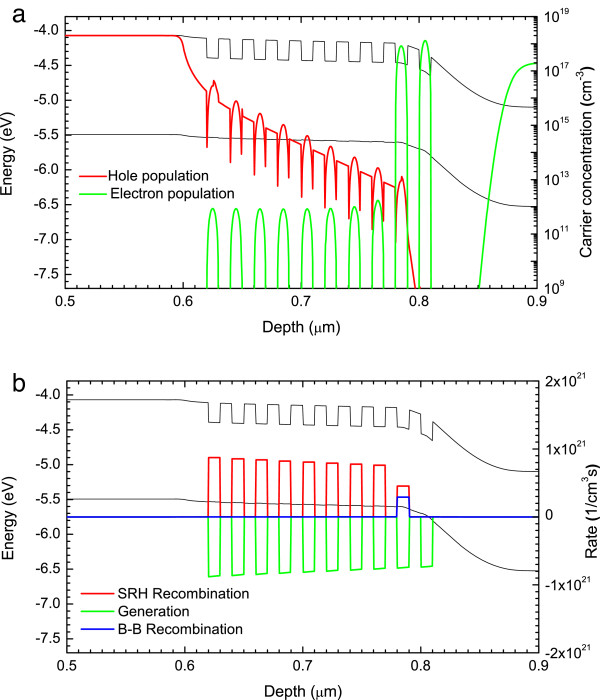
***V***_**app**_ **= 0.4 V.** Band profile and **(a)** electron/hole populations, **(b)** SRH, B-B recombination and optical generation rates.

We consider what happens to the band profile, carrier populations and recombination rates throughout the device when moving from forward to reverse bias, thus from the flat band conditions to increasing electric field. The modelled band profile and the electron and hole populations are shown in Figures 6a, 7 and 8a. The band profile, together with Shockley-Read-Hall (SRH), band-to-band (B-B) recombination and optical generation rates are shown in Figures 6b, 7 and 8b. The generation rate is shown to be negative for clarity, and the depth is measured from the top of the p-type region. In cases where few majority carriers enter the depletion region, the difference between the generation and recombination rate gives the number of carriers escaping from a well.

The first voltage considered is a forward bias of *V*_app_ = 1 V. At this bias, the total voltage drop across the device *V*_j_ is equal to 0.43 V (*V*_j_ = *V*_bi_ − *V*_app_). The resulting electric field occurs almost exclusively between QW1 and the beginning of n-type region, as shown by the band diagram in Figure 6a. The reason for the electric field being limited to this portion of the device is that a significant negative charge exists in QW1. This is due to majority of electrons in the n-type region being able to diffuse into QW1 at these low electric fields causing a large electron accumulation. As the electrons diffusing into QW1 are unlikely to escape, electron populations elsewhere in the intrinsic region are low. On the other hand, the hole populations are between 10^16^ and 10^13^ cm^−3^ for most of the intrinsic region, due to the low electric field at the p-i interface and to the poor hole confinement in the wells. The higher hole to electron populations in QW10 to QW2 will lead to a slight positive charge occurring in them, but not large enough to have a large impact on the devices performance. Figure 6b shows that the recombination rate is equal to the generation rate for QW10 to QW2; as with no electric field across these wells, the photogenerated electrons are unable to escape. For QW1, the recombination rate is slightly greater than the generation rate. This is due to both electrons and holes from the n- and p-type regions being able to diffuse into it and recombine in addition to the photogenerated carriers.

The next point voltage considered is *V*_app_ = 0.7 V, which lies at the highest point of the first peak (see Figure 5). Figure 7a shows clearly that almost all of the increase in the voltage is dropped between QW1 and the n-type region. This increase in electric field leads to the recombination rate (Figure 7b) dropping to less than half the generation rate in QW1, which corresponds to carriers escaping from the well. Consequently, PC increases when the applied voltage is reduced from 1 to 0.7 V.

The electron escape time will still be much larger than the hole escape time, resulting in the electron population in QW1 increasing compared to *V*_app_ = 1 V. While not clear from the band diagram, the electric field has slowly begun to be dropped across QW2 as well. This allows the poorly confined holes to escape causing the electron population in the QW2 to begin to increase and a negative charge develop.

At *V*_app_ = 0.4 V, which coincides with the minimum conductance between P1 and P2, the recombination rate for QW1 drops to zero (Figure 8b). This means that all carriers generated in QW1 are now escaping and contributing to the PC hence the conductance being zero. The negative charge and electron population in QW1 has dropped compared to their values at *V*_app_ = 0.7 V, as the higher electric field across the well decreases the electron escape time. At this bias, a significant electric field has developed across QW2. As was the case for QW1, any electric field across the well will cause the loosely confined holes to escape. This results in a high electron concentration hence a negative charge to develop in QW2. The oscillation that had led to the electrons escaping QW1 will now repeat for QW2 and eventually for every other QW in the device as the reverse bias is increased. This effect can be seen in the video included in the Additional file 1, which shows the evolution of the band energy diagram, the recombination rate and the charge and carrier distribution as a function of applied bias.

## Conclusions

In this paper, we investigated and modelled the PC oscillations observed in the low-temperature *I*-*V* characteristics of illuminated GaInNAs/GaAs MQW pin diodes. The number of the steps reflects the number of the QWs in the device. Modelling the devices using a semiconductor device simulation package shows that due to the low VB offset in dilute nitride material, the holes can escape from the wells much quicker than electrons resulting in the accumulation of negative charge in each well. This charge results in the electric field being applied one well at a time, and each step corresponds to the escape probability becoming low enough for photogenerated electrons to escape from a quantum well.

## Competing interests

The authors declare that they have no competing interests.

## Authors’ contributions

BR fabricated the investigated devices and performed the numerical simulation. The experimental work was done by BR and HK. Data analysis and manuscript conception were done by SM and BR. SM supervised the experimental work, and NB was the project supervisor. AE contributed to the discussion of the results and the writing of the manuscript. All authors read and approved the final manuscript.

## Supplementary Material

Additional file 1 **The video shows the modelling results achieved using Simwindows32 for sample AsN3134.** Four graphs are constantly updated as the applied voltage is swept from 1 to −5 V. The *x*-axis represents the distance from the top of the device, measured in μm. Precisely: top left, evolution of the band diagram, measured in eV, the green and red lines are the hole and electron Fermi levels, respectively; top right, total recombination rate, this is the recombination rate minus the generation rate in the units of cm^−3^ s^−1^; bottom left, total electron (blue) and hole (red) concentrations in the units of cm^-3^; bottom right, charge distribution in the units of C/cm^3^. Click here for file
